# Use of the Human Granulysin Transgenic Mice To Evaluate the Role of Granulysin Expression by CD8 T Cells in Immunity To Mycobacterium tuberculosis

**DOI:** 10.1128/mbio.03020-22

**Published:** 2022-11-21

**Authors:** Preeti Thakur, Rujapak Sutiwisesak, Yu-Jung Lu, Samuel M. Behar

**Affiliations:** a Department of Microbiology and Physiological Systems, University of Massachusetts Chan Medical School, Worcester, Massachusetts, USA; b Immunology and Microbiology Program, Graduate School of Biomedical Science, University of Massachusetts Chan Medical School, Worcester, Massachusetts, USA; c Princess Srisavangavadhana College of Medicine, Chulabhorn Royal Academy, Bangkok, Thailand; Weill Cornell Medical College

**Keywords:** CD8 T cells, *Mycobacterium tuberculosis*, antimicrobial peptides, granulysin, tuberculosis

## Abstract

The cytotoxic granules of human NK and CD8 T cells contain the effector molecule granulysin. Although *in vitro* studies indicate that granulysin is bactericidal to Mycobacterium tuberculosis and human CD8 T cells restrict intracellular M. tuberculosis by granule exocytosis, the role of granulysin in cell-mediated immunity against infection is incompletely understood, in part because a granulysin gene ortholog is absent in mice. Transgenic mice that express human granulysin (GNLY-Tg) under the control of human regulatory DNA sequences permit the study of granulysin *in vivo*. We assessed whether granulysin expression by murine CD8 T cells enhances their control of M. tuberculosis infection. GNLY-Tg mice did not control pulmonary M. tuberculosis infection better than non-Tg control mice, and purified GNLY-Tg and non-Tg CD8 T cells had a similar ability to transfer protection to T cell deficient mice. Lung CD8 T cells from infected control and GNLY-transgenic mice similarly controlled intracellular M. tuberculosis growth in macrophages *in vitro*. Importantly, after M. tuberculosis infection of GNLY-Tg mice, granulysin was detected in NK cells but not in CD8 T cells. Only after prolonged *in vitro* stimulation could granulysin expression be detected in antigen-specific CD8 T cells. GNLY-Tg mice are an imperfect model to determine whether granulysin expression by CD8 T cells enhances immunity against M. tuberculosis. Better models expressing granulysin are needed to explore the role of this antimicrobial effector molecule *in vivo*.

## INTRODUCTION

CD8 T cells are known as cytotoxic T lymphocytes (CTL) in recognition of their capacity to kill target cells. Killing of target cells proceeds by three mechanisms (1–3). The dominant pathway is cytotoxic granule exocytosis (4). Cytotoxic granules, which are found in the cytosol of CD8 T cells and NK cells, are specialized lysosomes that contain cytotoxins (e.g., granzymes). Upon CD8 T cell recognition of a target cell, the granules polarize to the immune synapse and release their contents. Cytotoxic granules contain perforin (PFN), which forms pores in the membranes of target cells, through which granzymes pass and induce death in the target cell. A second pathway is mediated by Fas ligand (FasL, CD95L), which is expressed on the surface of CTL (5). Upon binding to Fas (CD95), the extrinsic apoptosis pathway is activated in the target cell, leading to apoptosis. Finally, secretion of TNF by CTL can lead to cell death in TNF-sensitive target cells (6).

The process of cytotoxic degranulation occurs after CD8 T cell recognition of class I MHC-restricted antigens on target cells. Early after CD8 T cell activation, perforin and granzymes are released into the immunological synapse between the CD8 T cell and the target cell (7). A cytotoxic granular protein present in human and nonhuman primates, but not in mice, is a saponin-like pore forming protein called granulysin (8). Granulysin is a component of cytotoxic granules found in the cytosol of CD4 T cells, CD8 T cells, and NK cells. It is constitutively expressed in CD8 T cells as a 15 kDa protein, but after T cell activation, granulysin is cleaved to a 9 kDa cytotoxic form (9).

Multiple lines of evidence suggest that granulysin is important in the control of several intracellular pathogens, including Mycobacterium tuberculosis (10–13). The granulysin protein has antimicrobial activity when directly applied to M. tuberculosis bacilli and can kill drug-resistant M. tuberculosis strains (14). *In vitro* studies of human cells show that CTLs and NK cells expressing both granulysin and perforin are correlated with antimicrobial activity against Mycobacterium kansasii and M. tuberculosis (14, 15). Patients with active tuberculosis (TB) from Indonesia had significantly lower serum granulysin levels compared with healthy controls, and granulysin levels normalized after 2 months of treatment (16). Perforin and granulysin appeared to be depleted from CD8 cells in TB lung granulomas compared with uninfected control, raising the possibility that active TB granulomas develop when there is insufficient granulysin and perforin expression in diseased tissue (17). Analogously, granulysin-expressing T cells were more frequently found in tuberculoid (localized) leprosy lesions than in lepromatous (disseminated) leprosy lesions (18). CD8 T cells co-expressing granulysin, granzyme B (GzmB)., and perforin (i.e., tri-cytotoxic CD8 T cells), are linked to disease control. CD8 T cells make an important contribution to BCG-induced protection against TB in NHPs (19), and we surmise a role for granulysin because it is upregulated after BCG vaccination (20, 21). Such a role for granulysin-expressing CD8 T cells would also be consistent with their significant association with granuloma that restrict the growth of M. tuberculosis in the cynomolgus macaque TB model (22).

Given that mice lack the granulysin gene, *in vivo* studies to assess the contribution of granulysin to immunity against M. tuberculosis have not been possible. Instead, descriptive studies correlate the presence of granulysin-expressing cells with the outcome of TB. Human granulysin-transgenic (GNLY-Tg) mice are a small animal model that can be used to evaluate the role of granulysin *in vivo* (23). GNLY-Tg mice survive Toxoplasma gondii and Trypanosoma cruzi infections under conditions that are lethal to control mice (13). Here, we used GNLY-Tg mice to test the hypothesis that expression of granulysin endows CD8 T cells with a greater capacity to abrogate TB by killing intracellular M. tuberculosis.

## RESULTS

### GNLY-Tg and non-Tg mice control M. tuberculosis similarly.

**FIG 1:**
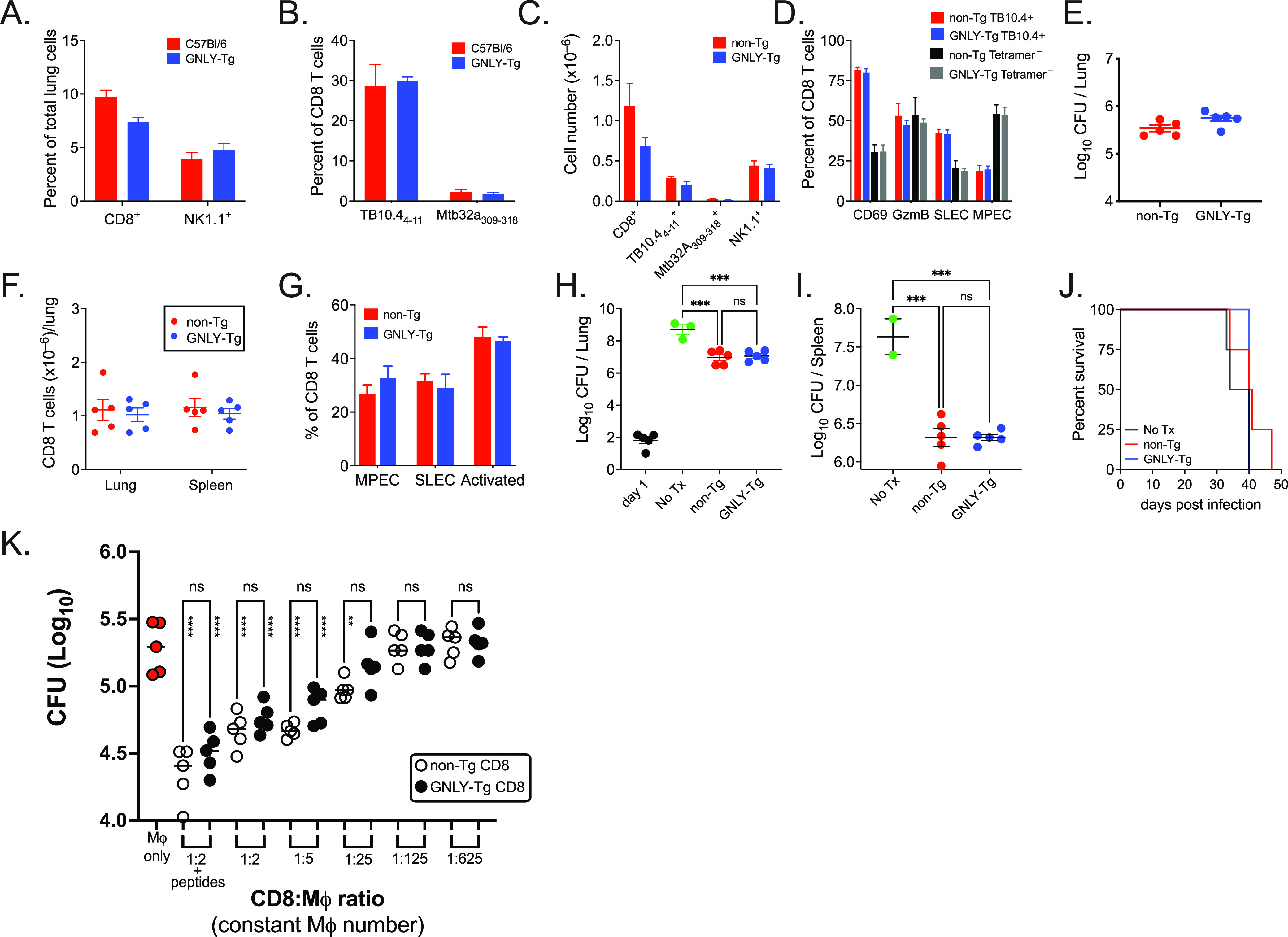
GNLY-Tg mice do not control of M. tuberculosis infection better than non-Tg mice. Control (C57BL/6, non-Tg) and GNLY-Tg mice were infected with M. tuberculosis, and after 16 weeks, the lung bacterial burden was determined, and flow cytometry was performed. (A) Proportion of CD8 T cells and NK cells in the lungs of non-Tg (red) and GNLY-Tg (blue) infected mice. (B) Proportion of tetramer positive cells among lung CD8 T cells from non-Tg (red) and GNLY-Tg (blue) infected mice. (C) Total numbers of pulmonary CD8 T cells, antigen-specific CD8 T cells, and NK cells. (D) Frequencies of CD8 T cells expressing CD69, GzmB, or having a SLEC or MPEC phenotype among GNLY-Tg (blue) and non-Tg (red) TB10.4 tetramer^+^ CD8 T cells, or non-Tg (black) or GNLY-Tg (gray) tetramer^–ve^ CD8 T cells. (E) Lung CFU from non-Tg littermate control or GNLY-Tg mice. (F to J) Purified CD8 T cells from GNLY-Tg or non-Tg mice were transferred to TCRα KO recipients 24 h prior to aerosol infection and analyzed 5 weeks later. (F) Total lung and spleen CD8 T cells, 5 weeks after transfer of TCRα KO mice with non-Tg (red) or GNLY-Tg (blue) CD8 T cells. (G) Frequencies of MPEC, SLEC, and activated CD8 T cells in the lungs of TCRα infected mice with transferred non-Tg (red) or GNLY-Tg (blue) CD8 T cells. Bacterial burden in the (H) lungs and (I) spleens of TCRα KO mice after transfer of CD8 T cells from non-Tg (red) or GNLY-Tg mice (blue). (J) Survival of M. tuberculosis-infected TCRα KO mice after transfer of CD8 T cells from non-Tg (red) or GNLY-Tg (blue) mice. (K) *In vitro* control of M. tuberculosis growth in macrophages by CD8 T cells isolated from the lungs of GNLY-Tg (filled) and non-Tg (open) infected mice at various effector:target ratios compared to macrophage only (red). (A to E) is representative data from five independent infections analyzed 4-, 8-, and 16-wpi, with *n* = 5 to 10 mice/group. (F to J) is data from one experiment each with *n* = 5/group. (K) is representative of two independent experiments, each using pooled cells from *n* = 5 mice/group. Statistical analysis was performed using a *t* test (A to G), one-way ANOVA (H, I), log-rank test (J), and two-way ANOVA (K). Comparison to infected macrophages is represented by vertical asterisks. **, *P* < 0.01; ***, *P* < 0.001; ****, *P* < 0.0001; ns, not significant.

To examine the importance of granulysin against *in vivo* control of M. tuberculosis infection, we infected GNLY-Tg and control mice (non-Tg, C57BL/6) with M. tuberculosis by the aerosol route. The mice were evaluated 4- and 16-weeks postinfection (wpi). There were no differences in the total number or frequency of total CD8 T cells, NK cells, or M. tuberculosis specific CD8 T cells (Fig. 1A to C; Fig. S1). Neither did we detect any differences in the frequency of CD69 or granzyme B-expressing CD8 T cells, or short-lived effector CD8 T cells (i.e., SLEC: KLRG1^+^CD127^–^), memory precursor effector CD8 T cells (i.e., MPEC: KLRG1^–^CD127^+^) (Fig. 1D). Importantly, the number of M. tuberculosis recovered from the lungs of GNLY-Tg mice and littermate controls was similar at 4- and 16-wpi (Fig. 1E).

As CD4 T cells have a dominant role in mediating protection in the murine model, we were concerned that CD4 T cells-mediated protection could obscure a difference between control and GNLY-Tg CD8 T cells. To focus on the contribution of CD8 T cells, we used an adoptive transfer model to eliminate the contribution of CD4 T cells. Highly purified splenic CD8 T cells from GNLY-Tg or littermate control (i.e., C57BL/6) mice were adoptively transferred to TCRα KO recipients, which were then infected with low-dose aerosolized M. tuberculosis (strain Erdman). After 35 days, similar numbers of CD8 T cells were detected in the lungs and spleen of TCRα ko mice that received GNLY-Tg or non-Tg CD8 T cells (Fig. 1F). There were no differences in the ability of GNLY-Tg or non-Tg CD8 T cells to differentiate into short-lived effector CD8 T cells (i.e., SLEC: KLRG1^+^CD127^–^), memory precursor effector CD8 T cells (i.e., MPEC: KLRG1^–^CD127^+^), or activated CD8 T cells (i.e., CD44^+^CD69^+^) (Fig. 1G). While CD8 T cells from both GNLY-Tg and non-Tg mice significantly protected TCRα knockout mice based on the M. tuberculosis bacillary burden, there was no difference between the groups (Fig. 1H and I). Neither genotype of CD8 T cells were able to extend the survival of TCRα knockout mice (Fig. 1J). Thus, despite the successful activation of CD8 T cells after transfer into TCRα knockout mice, GNLY-Tg CD8 T cells did not lead to better control of M. tuberculosis infection compared to non-Tg CD8 T cells.

We next asked whether CD8 T cells isolated from intact M. tuberculosis-infected GNLY-Tg and non-Tg mice differed in their ability to control intracellular M. tuberculosis in macrophages. Purified lung CD8 T cells from M. tuberculosis-infected GNLY-Tg and non-Tg mice were cultured at different effector to target ratios with a fixed number of infected macrophages. As previously observed, non-Tg CD8 T cells were able to control intracellular M. tuberculosis even at a CD8: macrophage ratio of 1:25 (Fig. 1K). However, there were no differences in the ability of GNLY-Tg and non-Tg CD8 T cells to restrict M. tuberculosis replication. Thus, these data show that neither GNLY-Tg mice nor their CD8 T cells were superior to non-Tg mice or their CD8 T cells in control of M. tuberculosis infection.

### Granulysin expression by activated CD8 T cells is not detected in the lungs of M. tuberculosis-infected GNLY-Tg mice.

**FIG 2:**
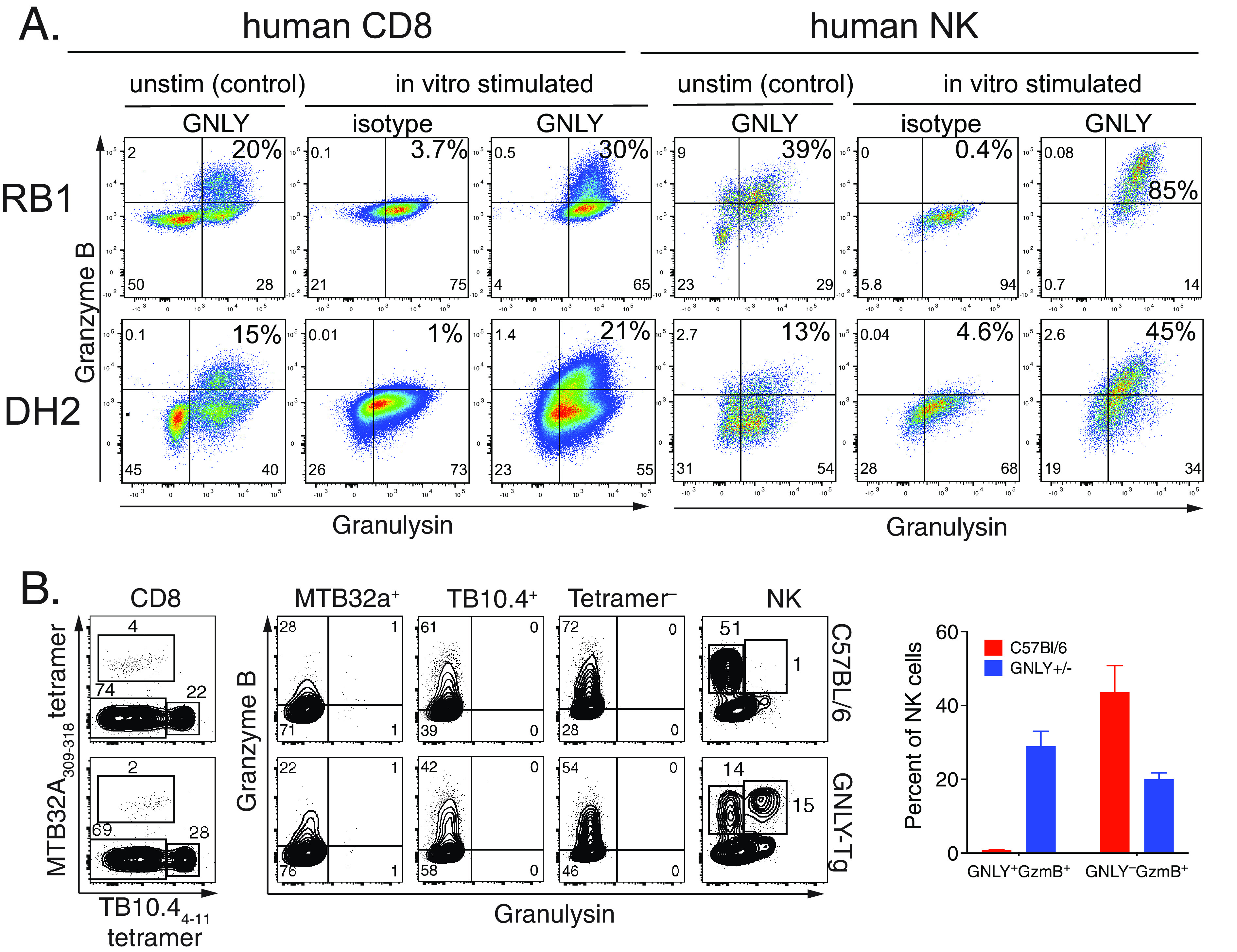
Granulysin is not expressed by CD8 T cells in the lungs of M. tuberculosis-infected GNLY-Tg mice. (A) Granulysin (GNLY) and granzyme B (GzmB) expression by human CD56^+^CD3^–^ NK and CD56^–^CD3^+^ CD8 T cells in unstimulated state (unstim) and 8 days after *in vitro* activation with anti-CD3/CD28 + IL-2. RB1 detecting total granulysin (upper row) and DH2 detecting 9 kDa (lower row). (B) Representative flow cytometry plots of granulysin and granzyme expression by NK cells, tetramer positive and tetramer negative CD8 T cells in non-Tg (upper panel) and GNLY-Tg (lower panel). Data representative data from five independent infections analyzed 4-, 8-, and 16-wpi, with *n* = 5 to 10 mice/group. (C) Expression of granulysin and granzyme B by NK cells in the lungs of M. tuberculosis-infected mice.

Given that granulysin has the potential to kill M. tuberculosis (14) and GNLY-Tg mice control other pathogens (24), we were surprised that GNLY-Tg mice did not control M. tuberculosis infection better than non-Tg mice. To confirm granulysin expression in the pulmonary compartment, we performed intracellular straining using two different human granulysin-specific antibodies, DH2 (25) and RB1 (26), which recognize the 9 kDa granulysin fragment and total granulysin, respectively. Human PBMC, after culture *in vitro* with or without stimulation by anti-CD3/anti-CD28 MAbs in the presence of IL-2 (or IL-15, data not shown), were used to verify that these anti-human granulysin MAbs detected intracellular granulysin by flow cytometry. As previously described, there are important differences in the ability of these two antibodies to detect human granulysin (Fig. 2A; Fig. S2). RB1 identified a well-defined population of CD8 T cells from unstimulated human PBMC that expressed granulysin (~20%), which co-expressed granzyme (Fig. 2A). Similarly, RB1 stains 40% to 50% of NK cells in PBMC. In contrast, the DH2 antibody identified fewer granulysin expressing NK and CD8 T cells in PBMC, which increased after stimulation (Fig. 2A).

**FIG 3:**
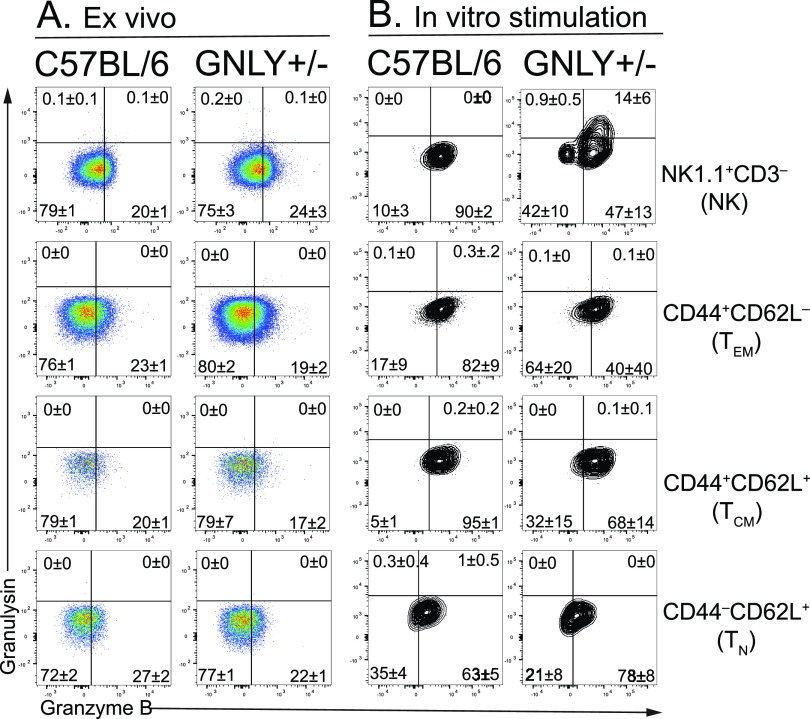
Granulysin is expressed in GNLY-Tg NK cells, but not CD8 T cells. (A, B) Lung mononuclear cells from non-Tg littermate control and GNLY-Tg mice were isolated from M. tuberculosis-infected mice 4 weeks after infection. The cells were either stained immediately (“A,” *ex vivo*) or after stimulation with TB10.4_4-11_ and 32A_309-318_ peptides and IL-2 (“B,” day 7, *in vitro*). Granulysin and granzyme expression by NK cells (CD3^–^NK1.1^+^) and CD8 T cells (CD3^+^CD8^+^NK1.1^–^) was determined by intracellular staining. The CD8 T cells were further divided based on expression of CD44 and CD62L as CD44^+^CD62L^–^ effector T cells (T_E_), CD44^+^CD62L^+^ central memory T cells (T_CM_), CD62L^+^CD44^–^ naive T cells (T_N_). Quadrants were based on FMO, isotype-matched antibody controls, and non-Tg littermates. Representative of three independent experiments each with four mice/group.

Using these anti-granulysin MAbs, we measured granulysin expression by lung CD8 T cells and NK cells from M. tuberculosis-infected GNLY-Tg mice and non-Tg controls 16-wpi. We used tetramers to identify TB10.4-specific and M. tuberculosis 32A-specific CD8 T cells, and the residual lung CD8 T cells (i.e., tetramer^–ve^). Analysis using RB1 showed that although the CD8 T cell populations were activated based on their expression of granzyme B, none of the cells expressed granulysin (Fig. 2B). NK cells were identified as CD3^–^ lymphocytes that expressed NK1.1. Half of the lung NK cells expressed granulysin and half of these expressed granzyme B (Fig. 2C). All the granulysin expressing cells co-expressed granzyme B. Analysis using the DH2 antibody did not identify granulysin expression by NK and CD8 T cells *ex vivo* at 4- or 16-wpi (Fig. 3; data not shown). Granulysin expression was not detected among B cells, neutrophils, alveolar macrophages, or CD4 T cells (Fig. S3). Thus, both the RB1 and DH2 MAbs failed to detect any evidence of granulysin expression by CD8 T cells in the lungs of M. tuberculosis-infected GNLY-Tg mice. We suggest that GNLY-Tg mice are not an appropriate model to study granulysin function in CD8 T cells during TB.

### Granulysin is poorly expressed by CD8 T cells from M. tuberculosis-infected GNLY-Tg mice.

Cells from the lungs and spleens of M. tuberculosis-infected GNLY-Tg and littermate controls were analyzed *ex vivo* or after *in vitro* activation (Fig. 3; Fig. S4). Little or no staining of NK or CD8 T cells was detected using the DH2 MAb, 4- or 16-wpi, indicating that the activation-induced form of granulysin could not be detected (Fig. 3A; data not shown). We considered the possibility that granulysin was not induced during the CD8 T cell response to M. tuberculosis. Therefore, we sought to identify conditions that led to upregulation of granulysin by CD8 T cells from GNLY-Tg mice (23). Huang et al. report that ~5% of CD8 and 33% of NK cells from GNLY-Tg mice express granulysin after stimulation with IL-15 (23); IL-2 and IL-15 are also known to induce granulysin in primary human CD8 T cells. To identify granulysin expression by antigen-specific T cells, lung cells from M. tuberculosis-infected mice 16-wpi were stimulated with TB10.4_4-11_ and 32A_309-318_ peptides, two immunodominant epitopes recognized by CD8 T cells epitopes, in media containing IL-2 for 8 days. Using the DH2 MAb, no granulysin expression was detected in stimulated CD8 T cells (Fig. 3B). In contrast, 14% of NK cells expressed granulysin after activation (Fig. 3B).

Splenocytes from these mice were analyzed in parallel. Nearly one third of the splenic NK cells expressed granulysin after *in vitro* culture. Also, expression of granulysin was detected by a small number of splenic CD8 T cells after their stimulation; more effector CD8 T cells expressed granulysin than other subsets (Fig. S4). Similar results were obtained after infection with Listeria monocytogenes in these mice (Fig. S5). Thus, despite using carefully controlled conditions, we could not detect *in vivo* granulysin expression by CD8 T cells in the lungs of GNLY-Tg mice during M. tuberculosis infection.

### Antigen-specific CD8 T cells can produce granulysin after repeated antigen stimulation.

**FIG 4:**
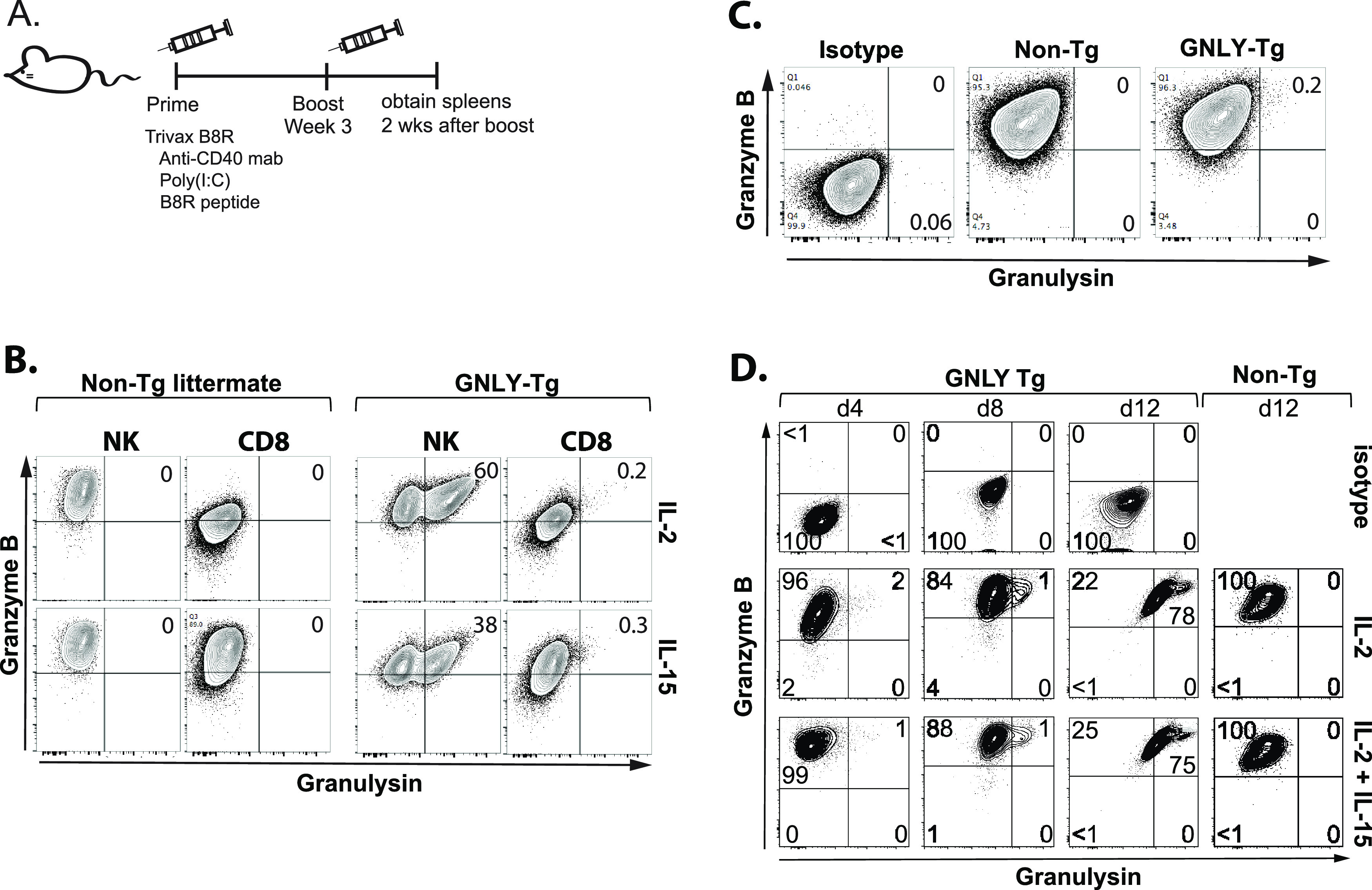
Granulysin expression by GNLY-Tg B8R_20-27_-specific CD8 T cell lines. (A) Schematic of strategy for generating GNLY-Tg B8R_20-27_-specific CD8 T cell lines. (B) Granulysin and granzyme B expression by NK or CD8 T cells was measured 5 days after *in vitro* stimulation of immune splenocytes with B8R peptide and cytokines (IL-2 or IL-15). (C) Splenic CD8 T cells were purified by negative selection and stimulated with B8R_20-27_-pulsed irradiated splenocytes and IL-2. Three weeks after stimulation, granulysin and granzyme B expression was measured. Performed once. (D) Non-Tg and GNLY-Tg B8R_20-27_-specific CD8 T cell lines were stimulated *in vitro* with B8R_20-27_-pulsed irradiated splenocytes and indicated cytokines. At indicated days post stimulations, granulysin and granzyme B expression were determined by flow cytometry. Gates by isotype controls. Performed twice using cell lines.

Although neither total nor 9 kDa granulysin expression was detected among M. tuberculosis-specific CD8 T cells in the lungs of infected mice, the activated form of granulysin can be expressed by splenic CD8 T cells after *in vitro* stimulation with anti-CD3 mAb and IL-2 (Fig. S4 and S5), albeit at low frequencies, indicating that it could potentially be expressed. To investigate granulysin expression, GNLY-Tg and non-Tg control mice were vaccinated with the B8R_20-27_ epitope from vaccinia, which elicits a substantial CD8 T cell response when using the Trivax immunization strategy (27) (Fig. 4A). Two weeks after the boost, splenocytes were stimulated *in vitro* with B8R_20-27_ peptide in the presence of IL-2 or IL-15. After 5 days, expression of granulysin was measured by RB1. Up to 60% of NK cells expressed both granzyme B and granulysin (Fig. 4B). Under these conditions, nearly all the CD8 T cells expressed granzyme B, but <0.5% expressed granulysin. Next, we derived CD8 T cell lines from these Trivax-B8R-vaccinated GNLY-Tg and non-Tg littermate controls by stimulating purified CD8 T cells with B8R-pulsed APC and IL-2. After 3 weeks in culture, B8R_20-27_-specific CD8 T cell lines from GNLY-Tg and C57BL/6 littermate control continued to express high granzyme B levels but did not express detectable granulysin (Fig. 4C). The B8R_20-27_-specific CD8 T cells were restimulated with B8R_20-27_-pulsed splenocytes in media supplemented with IL-2 or IL-15. Granulysin expression by CD8 T cells was detected by 8 days poststimulation (Fig. 4D). Like human CD8 T cells, IL-2 and IL-15 were strong stimulators of granulysin expression. Thus, GNLY-Tg CD8 T cells from spleen have the potential to express both total and activated form of granulysin after repeated stimulation in the presence of IL-2 or IL-15.

## DISCUSSION

T cells are crucial for the control of M. tuberculosis infection and CD4 T cells make an outsized contribution to immunity in the mouse TB model (28). CD8 T cells are necessary, but their overall contribution appears to be less than that for CD4 T cells. While the reasons for this hierarchy are unclear, recent data suggest that optimal CD8 function requires CD4 T cell help (29). Recent data from the NHP TB model have supported previous work that human CD8 T cells mediate protection against M. tuberculosis infection (6, 18, 19, 30–32). While the experimental approaches used in mice, NHP, and humans vary, another possibility is that functional differences exist among CD8 T cells from these different species. CTLs kill target cells by a variety of mechanisms, including cytotoxic granule exocytosis. Cytotoxic granules contain effector molecules, including antimicrobial peptides, which are directly toxic to intracellular pathogens. Granulysin kills several microbes, including Listeria monocytogenes, *Plasmodium yoelli*, Trypanosoma cruzi, Toxoplasma gondii, Leishmania major, and M. tuberculosis (10, 12–14). While granulysin is expressed by human and NHP CD8 T cells, the gene for granulysin is absent from rodents. Here, we hypothesized that the expression of granulysin by CD8 T cells would lead to improved control of M. tuberculosis infection.

GNLY-Tg mice were generated using a bacteria artificial chromosome system where the transgene was randomly integrated (23). In situ hybridization revealed the location of the transgene, and subsequent studies demonstrated the expression of granulysin RNA and protein. The Lieberman lab finds that GNLY-Tg mice have greater survival and decreased parasitemia compared to non-Tg mice after infection with T. cruzi and T. gondii (13). In this study, *in vivo* expression of granulysin protein by CD8 T cells from GNLY-Tg mice was not shown. Instead, an important role for granulysin is based on T cell depletion studies. While the overall impact of granulysin on the outcome of infection is impressive, the cellular mechanism is murky.

In the present study, no difference was detected in M. tuberculosis control between GNLY-Tg and non-Tg mice after low-dose aerosol infection, neither in intact mice nor in experimental approaches that used purified CD8 T cells. Importantly, we were unable to demonstrate granulysin expression in CD8 T cells, even when we used the granulysin-specific RB1 MAb, which detects total granulysin. Also, we did not detect total granulysin in CD4 T cells, B cells, neutrophils, and alveolar macrophages. NK cells in the lungs of M. tuberculosis-infected mice did express total granulysin *in vivo*, but not the activated form (i.e., 9 kDa), which is recognized by the DH2 MAb. However, contrary to *in vitro* studies in which human NK cells expressing both granulysin and perforin are correlated with antimicrobial activity against Mycobacterium kansasii and M. tuberculosis (15), granulysin expression by NK cells in GNLY-Tg mice did not lead to greater protection against M. tuberculosis infection compared with non-Tg mice.

GNLY-Tg mice did not express granulysin in the CD8 T cell compartment *in vivo*, which differs from what we and others have observed for human CD8 T cells in peripheral blood (18, 33). The lack of any evidence for granulysin expression by CD8 T cells *in vivo* using two different MAbs, RB1 and DH2, which detect total and the 9 kDa fragment of granulysin, respectively, impeded our effort to determine whether granulysin contributes to protection against M. tuberculosis infection *in vivo*. Nor could we detect expression of the activated form of granulysin by any lymphocytes in the spleen after L. monocytogenes infection. Despite the lack of evidence for granulysin expression *in vivo* by CD8 T cells, we could induce granulysin *in vitro* under a limited number of conditions. After stimulating lung cells or splenocytes for 7 days, we detected granulysin expression by a small number of NK cells and splenic CD8 T cells. The latter results are similar to studies from Walch et al.; however, the authors did not comment on whether they detected *ex vivo* expression of granulysin after L. monocytogenes infection (12). We suggest that the similar outcome of M. tuberculosis infection in GNLY-Tg and non-Tg mice results from a lack of granulysin expression by CD8 T cells from GNLY-Tg. Therefore, the ability of granulysin-expressing CD8 T cells to control M. tuberculosis infection cannot be assessed in this model.

Is it possible that the type of T cell response elicited by M. tuberculosis infection fails to induce granulysin expression? It is curious that *in vitro* stimulation induced activated granulysin in splenic but not lung CD8 T cells from M. tuberculosis-infected mice. Consistent with previous reports, we found that IL-2 and IL-15 induced granulysin in human and murine cells from uninfected donors (23, 33). Few pulmonary T cells produce IL-2 following M. tuberculosis infection, which could result in suboptimal activation of CD8 T cells and poor expression of their effector functions. Could M. tuberculosis infection lead to depletion of granulysin via cytotoxic granule exocytosis, as has been suggested for humans (17)? Both the 15 kDa and 9 kDa forms of granulysin can be secreted, but a substantial amount is intracellular (25). Although impaired perforin and granulysin expression might occur in chronic tuberculosis lesions in humans (17), our inability to detect granulysin produced by CD8 T cells from GNLY-Tg mice leads us to attribute this to a species difference. While some human CD8 T cells express granulysin even in an unstimulated state, CD8 T cells from these mice do not (23). Furthermore, although we were able to induce granulysin in antigen-specific CD8 T cells from uninfected (i.e., vaccinated) GNLY-Tg mice, we were able to so only after repeated *in vitro* stimulation with IL-2 and antigen. NK cells from hGNLY-Tg mice express the 15 and 9 kDa forms of granulysin protein at comparatively lower levels than unstimulated human NK cell, though upon stimulation have the capability to expresses both forms (23). As the kinetics and form of granulysin expressed by stimulated NK cells and CD8 T cells from GNLY-Tg mice appears to differ from human PBMC (23, 34), we suggest that the differences we observe are the result of incompatibility of the human regulatory sequences with murine transcription factors. In the future, it will be important to explore the change in granulysin expression pattern between NK cells and CD8 T cells from humans and GNLY-Tg mice. Such observations may lead to important insights in the regulation of granulysin expression and should allow the design of an improved mouse expressing human granulysin for future studies.

## MATERIALS AND METHODS

### Ethics statement.

Studies were conducted using the relevant guidelines and regulations and were approved by the Institutional Animal Care and Use Committee at the University of Massachusetts Medical School (UMMS) (Animal Welfare A3306-01), using the recommendations from the Guide for the Care and Use of Laboratory Animals of the National Institutes of Health and the Office of Laboratory Animal Welfare.

### Animals.

GNLY-Tg mice were obtained from Dr. Judy Lieberman (Children’s Hospital, Boston, MA) (13). The first batch of experiments were performed using with GNLY-Tg hemizygous mice that were bred to B6 controls to generate both GNLY-Tg (hemi) and littermate controls. A second batch of experiments used mice bred from homozygous GNLY-Tg female and GNLY-Tg male mice. Non-Tg (i.e., wild-type C57BL/6) littermate mice were used as controls. C57BL/6J were purchased from Jackson Laboratories (Bar Harbor, ME). TCRα knockout mice were obtained from Jackson Laboratories and bred locally. All mice were housed under specific pathogen-free conditions at UMMS. Mice were 8 to 9 weeks old at the start of all experiments. Infected mice were housed in biosafety level 3 facilities under specific pathogen-free conditions at UMMS.

### Mouse infections.

Eight- to 9-week-old female mice were infected by the aerosol route as described previously (27, 35). The M. tuberculosis Erdman strain was used for *in vivo* infections. Frozen bacterial stocks were thawed, diluted in 0.9% NaCl with 0.02% Tween80, and sonicated before loading into a nebulizer for Glas-Col aerosol chamber (Glas-Col LLC, Terre Haute, IN) to deliver approximately 100 CFU to the lungs of each mouse. The infecting dose was determined 16 h after infection by plating lung homogenates on 7H11 agar plates (Hardy Diagnostics). Lungs and spleens were aseptically removed, individually homogenized, and plated to determine viable bacteria (27, 35). For infection with Listeria monocytogenes, 8-week-old female mice were injected intraperitoneally with Listeria strain 10403s: 2 × 10^4^ bacteria in PBS (36). Seven days later, spleens were isolated aseptically, processed for single cell suspension, and stimulated with *in vitro* stimulated with anti-CD3/CD28 MAb and IL-2.

### Preparation of lung cells.

Mice were euthanized, lungs perfused with 10 mL of cold RPMI 1640, and lung single cell suspensions prepared using a combination of GentleMACS tissue dissociator (Miltenyi Biotec, Germany) and enzymatic digestion (27).

### Adoptive transfer experiment.

CD8 T cells were purified from spleens and lymph nodes by performing an immunomagnetic negative (untouched) selection using EasySep Mouse CD8^+^ T Cell isolation kit (STEMCELL Technologies), with a resulting purity of 90% to 95%. Five-million purified CD8 T cells were transferred intravenously to each recipient TCRα knockout mouse, and the mice were infected by the aerosol route within 24 h.

### *In vitro* stimulations.

Single cells suspension from the spleens of Listeria infected mice were stimulated *in vitro* in 24-well culture plates with anti-CD3 MAb, clone 145-2C11 from Biolegend (1 μg/mL) and anti-CD28 MAb, clone 37.51 from Biolegend (0.5 μg/mL) plus IL-2 (50 U/mL) or with IL-2 only. Lung or spleen single cell suspensions from M. tuberculosis-infected mice were stimulated with TB10.4_4-11_ and 32A_309-318_ peptides (10 uM) in solution plus IL-2 (50 U/mL). In some experiments, the combination of soluble anti-CD3/CD28 MAbs was used instead of peptides. Media was replenished with complete RPMI cRPMI; 10% heat-inactivated FCS, 10 mM HEPES, 1 mM sodium pyruvate, 2 mM l-glutamine, 50 mg/mL streptomycin, and 50 U/mL penicillin (all from Invitrogen), 0.05 mM 2-mercaptoethanol (Gibco) and IL-2 (50 U/mL) every third day. Cells were analyzed by flow cytometry after 7 days.

### Human cells.

Human PBMCs were isolated from peripheral blood or leukopaks and stimulated *in vitro* with anti-human CD3 (Clone OKT3) and anti-human CD28 (Clone CD28.2) Antibodies in solution, with recombinant human IL-2/IL-15. After 8 days, cells were stained for granulysin and granzyme B expression as described below.

### Derivation of B8R_20-27_-specific CD8 T cell lines.

The B8R peptide (B8R_20-27_ from vaccinia) was synthesized by New England Peptides (Gardner, MA). GNLY-Tg or non-Tg littermate controls were vaccinated with B8R_20-27_ using the Trivax strategy (27, 37). Three weeks after the final boost, single cell suspensions from spleens and LNs were stimulated in the presence of cytokines (35). Cytokines were used at the following concentrations: IL-2 (50 U/mL), IL-7 (5 ng/mL), IL-15 (50 ng/mL), and IL-21 (50 ng/mL) (all from Peprotech).

### Flow cytometry.

Mouse cells were stained with Zombie Violet or Aqua Fixable viability dye, and the antibodies to: CD19 (6D5), FITC-CD3ε (Clone 145-2C11), CD4 (GK1.5), PerCP/cyanine 5.5-CD8a (Clone 53-6.7), BV421-NK1.1 (Clone PK136), APC/cyanine 7 CD44 (Clone IM7), PE/Cyanine 7 CD62L (Clone MEL-14), CD127 (A7R34), KLRG1 (2F1/KLRG1), CD69 (H1.2F3), PE anti-human Granulysin (DH2), Alexa fluor 647 anti-human/mouse Granzyme B (GB11) (all from Biolegend), and/or Alexa Fluor 488 anti-human granulysin (RB1; BD biosciences), B8R, TB10.4-specific and Mtb32A-specific tetramers were obtained from the National Institutes of Health Tetramer Core Facility (Emory University Vaccine Center, Atlanta, GA). Human cells were stained using (Zombie Violet/Aqua) Fixable viability dye, and antibodies to: PE/Cyanine 7-CD3ε (Clone UCHT1), FITC-CD8a (Clone SK1), CD56 (Clone HCD56), PE anti-human Granulysin (DH2), Alexa fluor647 anti-human/mouse Granzyme B (GB11) (all from Biolegend). and/or Per CP/Cyanine 5.5-CD8a (SK1), and Alexa Fluor 488 anti-human granulysin (RB1; BD biosciences). The cells were permeabilized with Cytofix/Cytoperm buffer from BD Biosciences (cat 554772) for 30 min at 4°C followed by wash with 1× BD perm/wash (cat 554723) before intracellular stain. Samples were fixed with 1% paraformaldehyde in PBS for >1 h. before analysis with a MACSQuant flow cytometer (Miltenyi Biotec). FlowJo Software (Tree Star, Portland, OR) was used for data analysis. Single lymphocytes were gated by forward scatter versus height and side scatter for size and granularity, and dead cells were excluded.

### Statistical analysis.

Data are represented as mean ± standard error of the mean (SEM). A two-tailed, unpaired Student's *t* test was used to compare two groups; a one-way ANOVA was used for >2 groups. A *P* value < 0.05 was considered significant. Analyses were performed using Prism (GraphPad Software, La Jolla, CA).

10.1128/mbio.03020-22.1 FIG S1 Flow cytometry gating strategy for the analysis of murine lung NK and T cells. Lymphocytes were identified using FSC and SSC properties, and dead cells were excluded by their uptake of Zombie viability dyes. Doublets were excluded by their nonlinear FSC-H and FSC-A properties. NK cells and non-NK cells were distinguished using the lineage markers NK1.1 and CD3. NK cells were identified as CD56^+^CD3^–^. CD8 T cells identified using the lineage marker CD8a and CD3, and antigen-specific CD8 T cells were identified using tetramers. Here, as an example, TB10.4-specific CD8 T cells were identified using H-2 K^b^ tetramers loaded with TB10.4_4-11_ peptide. Download FIG S1, PDF file, 0.3 MB. Copyright © 2022 Thakur et al. 2022 Thakur et al. https://creativecommons.org/licenses/by/4.0/ This content is distributed under the terms of the Creative Commons Attribution 4.0 International license.

10.1128/mbio.03020-22.2 FIG S2 Flow cytometry gating strategy for the analysis of human peripheral blood NK and T cells. Dead cells were excluded by their uptake of Zombie viability dyes and lymphocytes were identified using FSC and SSC properties. Doublets were excluded by their nonlinear FSC-H and FSC-A properties. NK cells and T cells were distinguished using the lineage markers CD56 and CD3. NK cells were identified as CD56^+^CD3^–^. CD8 T cells identified using the lineage marker CD8a and CD3. Corresponding fluorescence minus one (FMO) were used as controls to define gating for NK and CD8 cells. Download FIG S2, PDF file, 0.4 MB. Copyright © 2022 Thakur et al. 2022 Thakur et al. https://creativecommons.org/licenses/by/4.0/ This content is distributed under the terms of the Creative Commons Attribution 4.0 International license.

10.1128/mbio.03020-22.3 FIG S3 Flow cytometry gating strategy for other cell populations in the murine lung. (A) Cells were identified using FSC and SSC properties, and doublets were excluded by their nonlinear FSC-H and FSC-A properties. Dead cells were excluded by their uptake of Zombie viability dyes. Leukocytes were identified based on expression of CD45. B cells were identified using the lineage marker CD19. From the population of non-B cells, NK cells and T cells were distinguished using the lineage markers NK1.1 and CD3. Activated T cells were identified by their expression of CD44, and CD4 and CD8 T cell subsets were recognized. From the non-B cell population, alveolar macrophages and eosinophils were distinguished based on their differential expression of CD11c and SiglecF. Finally, neutrophils were identified as Ly6G^+^. (B to D) After gating, all cell types were analyzed for intracellular expression of granulysin using gates based on staining with an isotype control. A representative C57BL/6 (non-Tg control) and GNLY-Tg mouse is shown for (B) alveolar macrophages, (C) neutrophils, (D) B cells, and (E) CD4 T cells. Download FIG S3, PDF file, 0.8 MB. Copyright © 2022 Thakur et al. 2022 Thakur et al. https://creativecommons.org/licenses/by/4.0/ This content is distributed under the terms of the Creative Commons Attribution 4.0 International license.

10.1128/mbio.03020-22.4 FIG S4 Granulysin expression *ex vivo* and after *in vitro* stimulation in GNLY-Tg splenic NK and CD8 T cells post-M. tuberculosis infection. Splenocytes from non-Tg littermate control and GNLY-Tg mice were isolated from M. tuberculosis-infected mice 4 weeks after infection. The cells were either stained immediately (*ex vivo*) or after stimulation with anti-CD3/CD28 MAbs and IL-2 (day 7, *in vitro*). Granulysin and granzyme B expression by NK cells (CD3^–^NK1.1^+^) and CD8 T cells (CD3^+^CD8^+^NK1.1^–^) was determined by intracellular staining. The CD8 T cells were further divided based on expression of CD44 and CD62L. Quadrants were based on FMO, isotype-matched antibody controls, and non-Tg littermates. Representative of three independent experiments each with four mice/group. Download FIG S4, PDF file, 0.8 MB. Copyright © 2022 Thakur et al. 2022 Thakur et al. https://creativecommons.org/licenses/by/4.0/ This content is distributed under the terms of the Creative Commons Attribution 4.0 International license.

10.1128/mbio.03020-22.5 FIG S5 Granulysin expression *ex vivo* and after *in vitro* stimulation in GNLY-Tg splenic NK and CD8 T cells post-Listeria monocytogenes (Lm) infection. Granulysin expression in spleens of Lm-infected mice. Splenocytes from non-Tg littermate controls and GNLY-Tg mice were analyzed for granulysin expression 7 days after infection. The cells were stained directly (*ex vivo*) or after stimulation with anti-CD3/CD28 MAbs and IL-2 for 7 days (*in vitro*). Expression of granulysin and granzyme B was analyzed in NK cells (CD3-NK1.1^+^) and CD8 T cells (CD3^+^CD8^+^NK1.1^–^) by intracellular staining. The CD8 T cells were categorized into different groups with CD44 and CD62L staining as CD44^+^CD62L^–^ effector T cells (T_E_), CD44^+^CD62L^+.^central memory T cells (T_CM_), CD62L^+^CD44^–^ naive T cells (T_N_). Quadrants were drawn based on FMO, isotype matched antibody and non-Tg littermate controls. Experiment is representative of two independent experiments each with three mice per group. Download FIG S5, PDF file, 0.5 MB. Copyright © 2022 Thakur et al. 2022 Thakur et al. https://creativecommons.org/licenses/by/4.0/ This content is distributed under the terms of the Creative Commons Attribution 4.0 International license.
