# Supplementary figures and images for: Use of the Human Granulysin Transgenic Mice To Evaluate the Role of Granulysin Expression by CD8 T Cells in Immunity To Mycobacterium tuberculosis
Source: mBio. 2022 Nov 21;13(6):e03020-22. doi: 10.1128/mbio.03020-22 (PMC9765553; doi:10.1128/mbio.03020-22)

## Gating strategy for murine lung

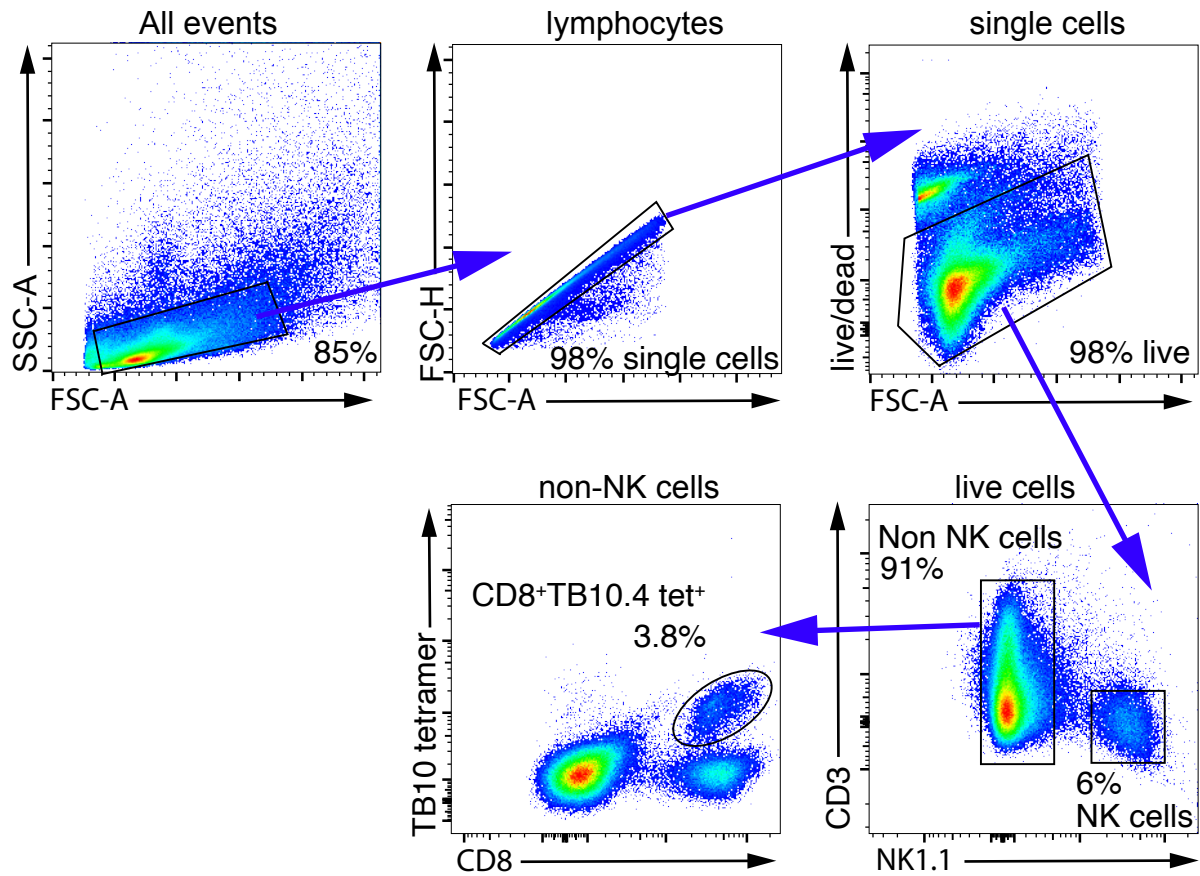

Supplement: FIG S1 [file mbio.03020-22-s0001.pdf]

## Gating strategy for human PBMC

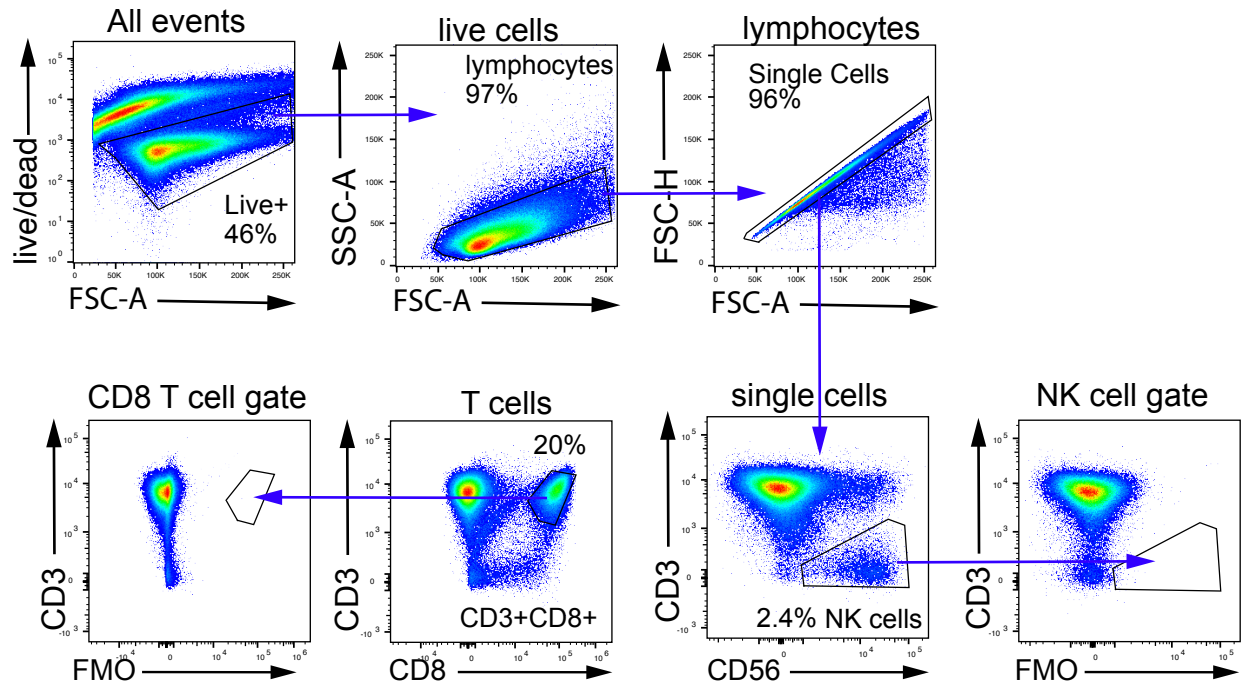

Supplement: FIG S2 [file mbio.03020-22-s0002.pdf]

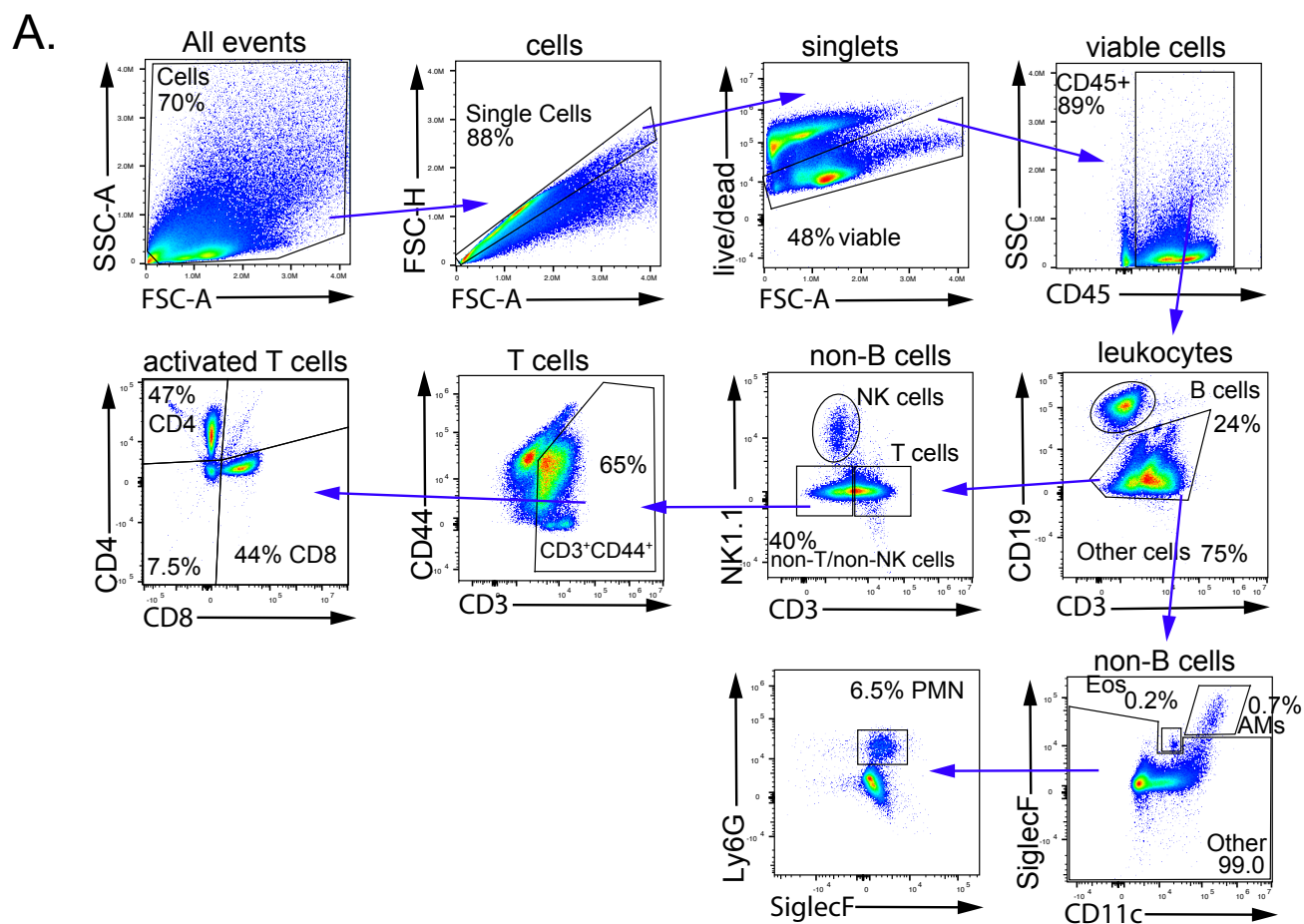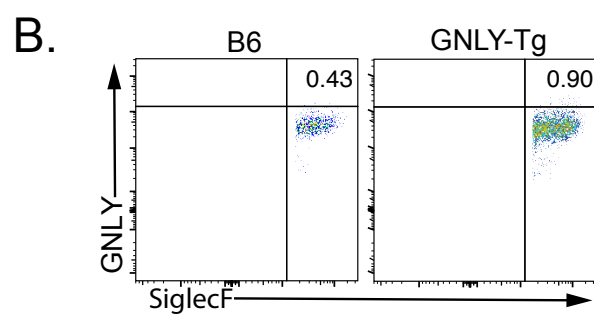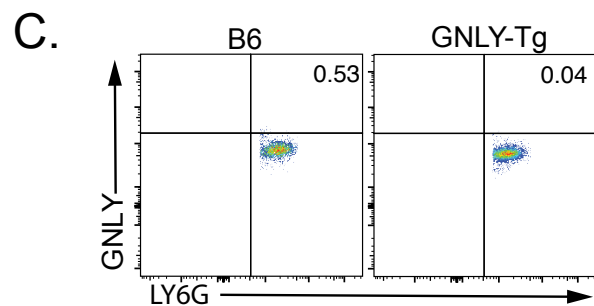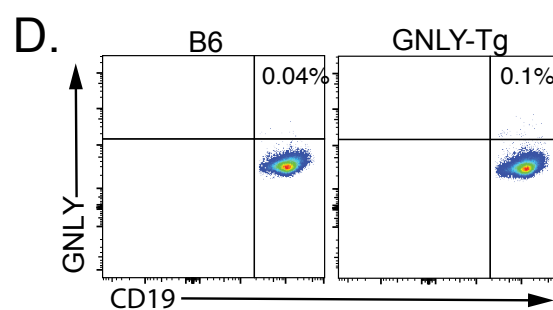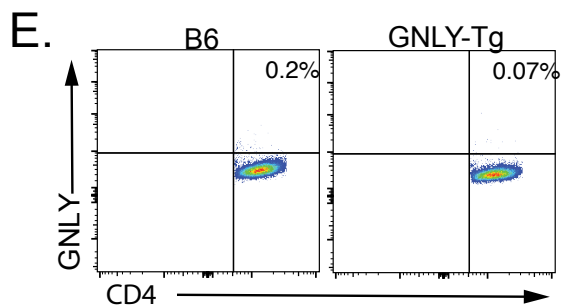

Supplement: FIG S3 [file mbio.03020-22-s0003.pdf]

Ex vivo

In vitro

C57BL/6

GNLY+/-

C57BL/6

GNLY+/-

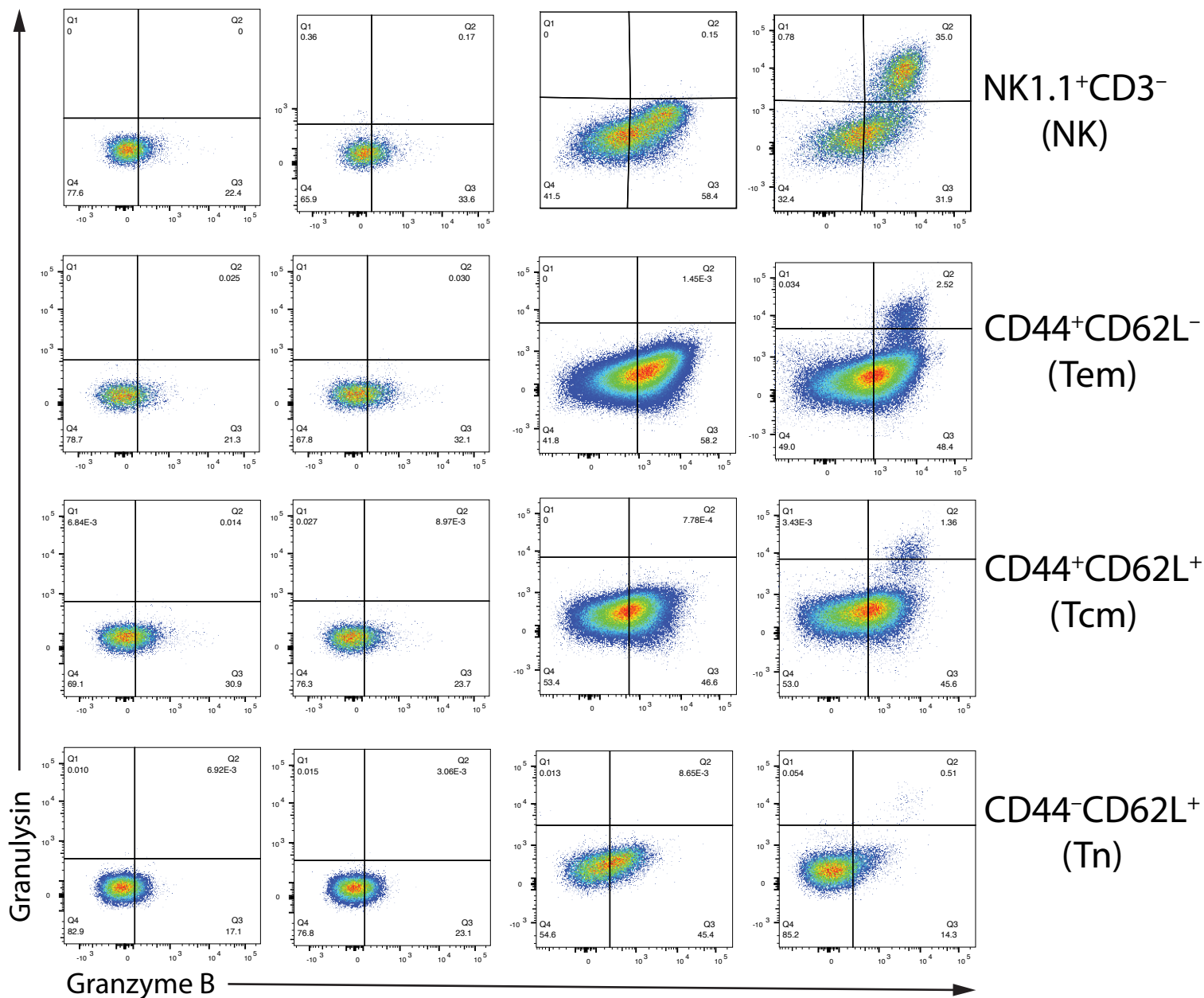

Supplement: FIG S4 [file mbio.03020-22-s0004.pdf]

# Listeria monocytogenes infection (in vitro stimulation)

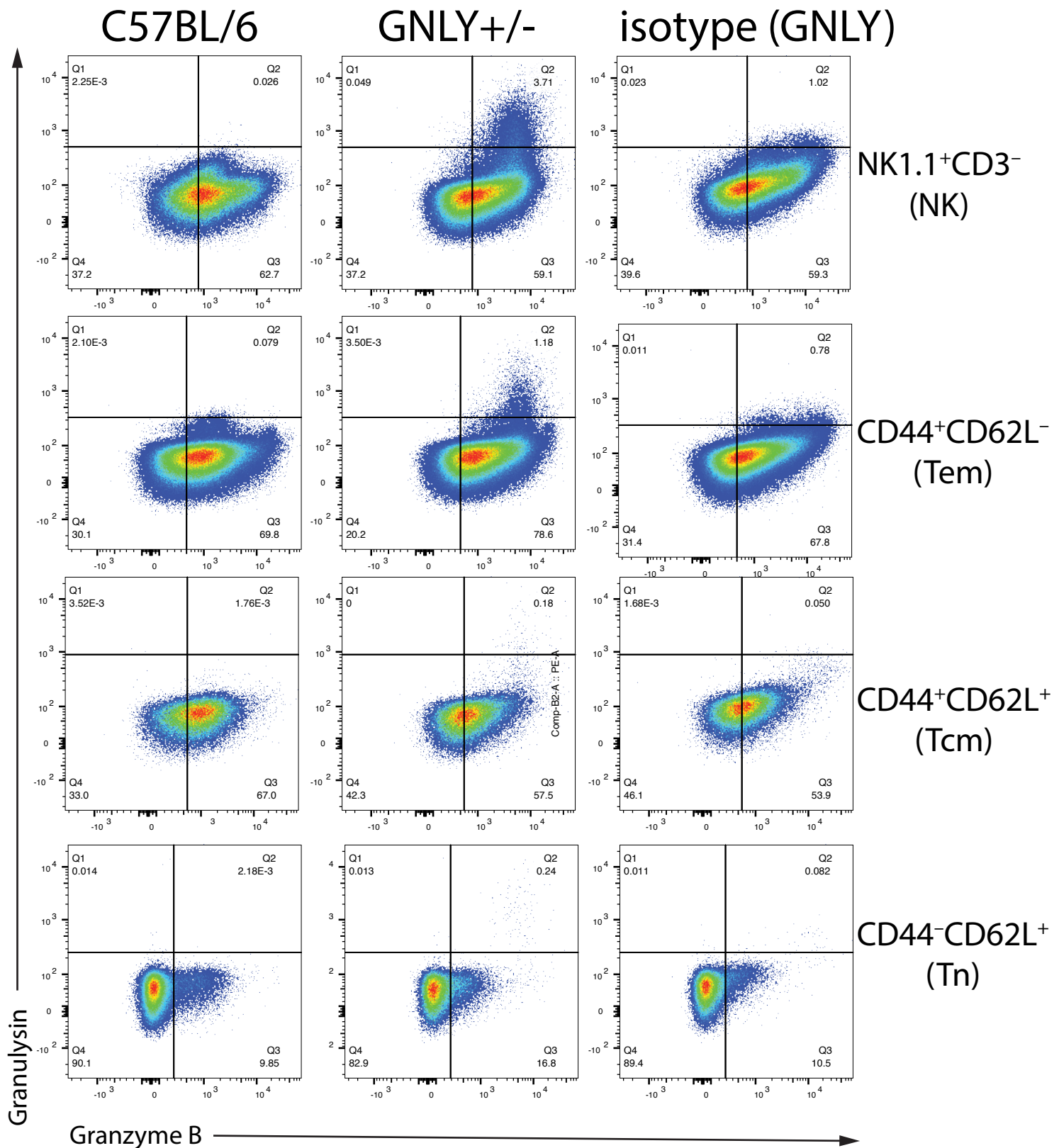

Supplement: FIG S5 [file mbio.03020-22-s0005.pdf]
